# Clinical Significance of T-Cell Immunoglobulin Mucin 3 Expression on Peripheral Blood Mononuclear Cells in Pediatric Acute Immune Thrombocytopenia

**DOI:** 10.1177/1076029617730638

**Published:** 2017-10-04

**Authors:** Asmaa M. Zahran, Mervat A. M. Youssef, Khalid I. Elsayh, Mustafa M. Embaby, Ahmad I. M. Ibrahim

**Affiliations:** 1 Clinical Pathology Department, South Egypt Cancer Institute, Assiut University, Assiut, Egypt; 2 Pediatric Department, Faculty of Medicine, Assiut University, Assiut, Egypt

**Keywords:** mononuclear cells, T-cell immunoglobulin mucin 3, immune thrombocytopenia

## Abstract

T-cell immunoglobulin mucin 3 (TIM-3) is a transmembrane protein that plays an important role in several autoimmune diseases. The relationship between TIM-3 and excessive immune responses in immune thrombocytopenia (ITP) is still unknown. In this study, we evaluated the relationship between the expression of TIM-3 on peripheral blood mononuclear cells in patients with ITP and the disease severity. The frequency of lymphocyte and monocyte subsets and their TIM-3 expression were evaluated in patients with acute ITP (n = 45) and in healthy control (n = 20) using flow cytometry. Based on bleeding severity, patients were classified into 3 subgroups as mild (n = 12), moderate (n = 25), and severe (n = 8) bleeding. T-helper lymphocytes was found to be significantly decreased in the severe bleeding group compared to the mild and moderate bleeding groups, while CD56^high^ natural killer (NK) cells were significantly expanded in severe bleeding group. In contrast, classical, intermediate, and nonclassical monocytes, natural killer T lymphocyte (NKT), and CD56^dim^ NK cells showed no significant changes among different patient groups. This alteration of lymphocyte and monocyte subsets was associated with significant decrease in TIM-3 expression on CD56^high^ NK cells, T-helper lymphocytes, NKT cells, and nonclassical monocytes in patients with ITP compared to the controls. Lower level of TIM-3 was found in severe bleeding group compared to mild and moderate bleeding groups. These results indicate that TIM-3 may be involved in the pathogenesis of ITP which subsequently can represent an opportunity for new therapeutic plan, moreover. This may have a prognostic value for disease severity.

## Introduction

Immune thrombocytopenia (ITP) is an autoimmune disorder characterized by isolated thrombocytopenia (platelet count in peripheral blood <100 × 109/L) with no other causes or disorders that can be associated with thrombocytopenia.^1^ The ITP is caused by immune dysregulation involving different mechanisms. These mechanisms include formation of antiplatelet antibodies of the immunoglobulin G (IgG) type against several platelet surface antigens,^2^ complement fixation,^3^ and dysfunction of T lymphocytes, which plays important roles in the pathophysiology of ITP.

Many of the features of T-lymphocyte dysregulation have been described in ITP, such as the shift in T–helper 1/T-helper 2 (Th1/Th2) balance,^4^ increased Th17,^5^ and decreased numbers of CD4^+^ CD25^+^ T-regulatory cells, which function to downregulate the T-cell responses.^6^ Moreover, cytotoxic T lymphocyte (CD8^+^) can mediate platelet destruction,^7,8^ as platelet-specific CD8^+^ have been found to be elevated in patients with active ITP.^9^ Additionally, the ratio between T-helper lymphocyte (CD4^+^) and CD8^+^ (CD4:CD8) is diminished in ITP and improves with disease remission.^10^

The natural killer (NK) cell is another T-lymphocyte subset with regulatory functions. There are 2 distinct NK cell subsets based on the level of CD56 and CD16 coexpression: CD56^high^ CD16^+^ NK and CD56^dim^ CD16^+^ NK. The CD56high CD16^+^ NK cells produce abundant interferon gamma (IFN-γ), while CD56^dim^ CD16^+^ NK cells are more cytolytic^11^ and found to be decreased in patients with ITP.^12^ The role and phenotype of NK in the autoimmune processes of ITP is still unclear.^13^

Natural killer T-lymphocyte (NKT) cells are associated with multiple human autoimmune diseases; however, the mechanisms are not yet clear.^14^ There is a controversy over the level of the NKT in ITP; some studies recorded an elevated NKT level,^15,16^ while others demonstrated a decreased NKT level in patients with ITP.^17^

Monocytes is the largest type of leukocyte and play an important role in autoimmune diseases. Based on CD14 and CD16 coexpression, there are 3 types of monocytes in human blood: classical (CD14^+^ CD16^−^), nonclassical (CD14^+^ CD16^++^), and intermediate (CD14^++^ CD16^+^). Classical monocyte has a phagocytic activity and nonclassical monocyte produce higher tumor necrosis factor (TNF) after stimulation.^18^ The nonclassical monocyte subset has been suggested to play different roles in the pathogenesis of ITP.^19^

T-cell immunoglobulin mucin 3 (TIM-3) is a type I transmembrane protein that has been expressed at high level on Th1 cells^20^ and low level in Th17 cell.^21^ It is also expressed on dendritic cells, monocytes,^22–24^ CD8^+^ T cells,^25,26^ and NK cells.^27^ It was found to be an important negative regulator of Th1 cell that inhibits aggressive Th1-mediated immune responses^28,29^ and a regulator of Th1 and Th17 cytokine secretion.^18^ The expression of TIM-3 on human T cells regulates cell proliferation and IFN-γ secretion.^14,30,31^ It interacts with galectin 9 that induce death of TIM-3 bearing cells.^32^ As the relationship between TIM-3 and excessive immune responses in ITP remains unclear, we investigated the expression of TIM-3 on peripheral blood mononuclear cells (PBMCs) and evaluated the contribution of TIM-3 expression in the severity of ITP in children with active disease.

## Patients and Methods

Forty-five children with acute ITP in addition to 20 healthy children as a control group were enrolled in this case control study. The study was carried in the Pediatric Hematology Unit of Children Hospital, Assiut University, between August 2016 and January 2017. The patients were 21 females and 24 males with mean age of 5.7 (3.4) years and a range of 2.9 to 11.4 years. All patients were at the onset of the acute disease. Diagnosis of ITP was based on the presence of thrombocytopenia (platelet count less than 100 × 109/L) with no evidence of other blood cell abnormalities, absence of organ enlargement, and exclusion of other known causes of thrombocytopenia, such as connective tissue disease, malignancy, drug-induced thrombocytopenia, and congenital thrombocytopenia.^33^ Patients with secondary or congenital thrombocytopenia were excluded.

At diagnosis, a full history of all patients and controls were recorded, including disease duration, drug intake, preceding viral infection, and bleeding manifestations. The full clinical examination was conducted, stressing on organomegaly and lymphadenopathy. Blood samples were taken from all patients on admission, then they were managed immediately according to our unite protocol. Patients did not receive any steroid or immunosuppressive drugs before sampling to avoid their effect on surface markers of lymphocytes.^34^ Patients in this study were classified into 3 subgroups according to the severity of bleeding.^35^

Group 1 (mild bleeding): Petechiae less than or equal to 10 in a patient’s palm-sized area, ecchymoses were 3 or more in the same body area, oral cavity hemorrhagic bullae or blisters (less than 3 or any number if reported by the patient), or epistaxis lasting ≤5 minutes.
Group 2 (moderate bleeding): Oral cavity bleeding from lips and tongue bites or after deciduous tooth loss or hemorrhagic bullae or blisters (from 3 to 10 but no difficulty with mastication), macroscopic hematuria (or described in a medical report), mild gastrointestinal tract (GIT) bleeding (present at the visit or described in a medical report), or epistaxis lasting >5 minutes.
Group 3 (severe bleeding): Epistaxis need packing or cauterization, intracranial bleeding, or any internal bleeding as hemoperitoneum, hemopericardium, hemothorax, macroscopic hematuria (requiring cystoscopy or other therapeutic procedures), or severe GIT bleeding (requiring endoscopy or other therapeutic procedures).

**Figure 1.**
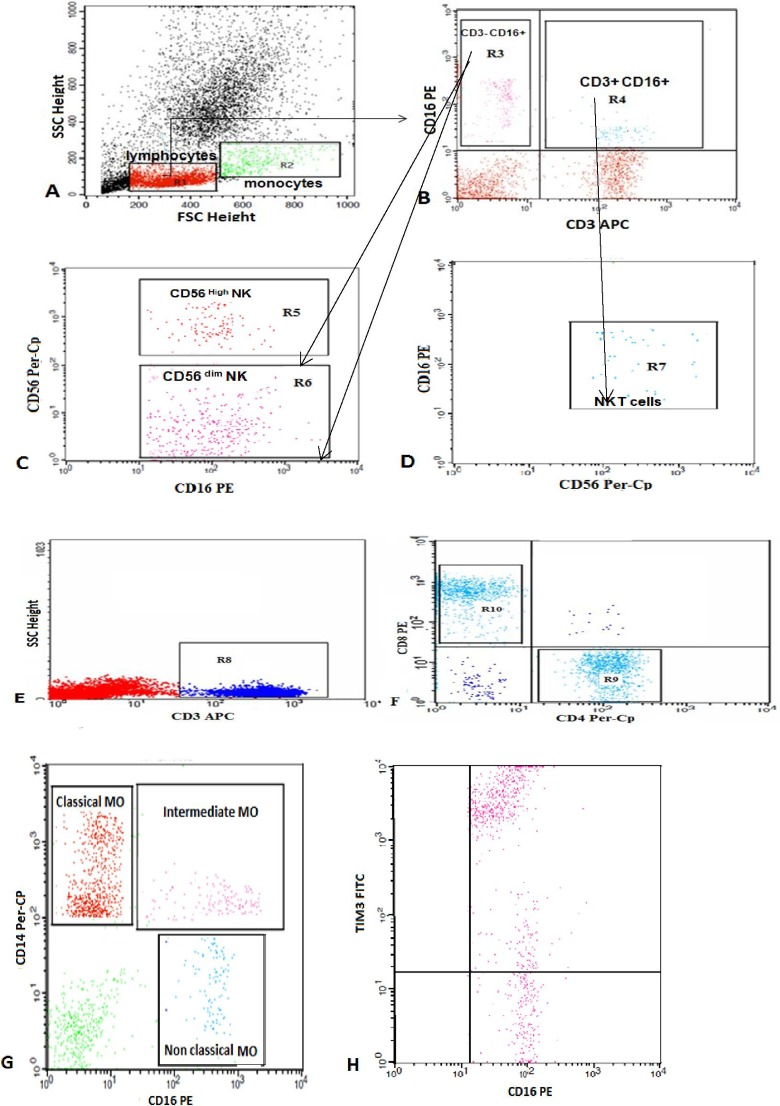
Flow cytometric detection of lymphocyte and monocyte subsets and their expression of TIM-3. A, Forward and side scatter histograms were used to define the monocyte and lymphocyte populations. B, C, and D, The expression of CD3, CD16, and CD56 were assessed in the lymphocyte population to detect NKT cells (CD3^+^ CD16^+^ CD56^+^), CD3^−^ CD16^+^ CD56^+high^ NK cells, and CD3^−^ CD16^+^ CD56^+dim^ NK cells. E and F, CD3^+^ cells (T lymphocytes) were then gated and the expression of CD4 and CD8 in the T lymphocytes was assessed to detect CD4^+^ (T-helper cells) and CD8^+^ (T-cytotoxic cells). G, The expression of CD14 and CD16 were analyzed on monocyte population. The classical monocytes (CD14^++^ CD16^−^), intermediate monocytes (CD14^+^ CD16^+^), and nonclassical monocytes (CD14^−/dim^ CD16^+^) were gated. H, The expression of TIM-3 in classical monocytes as an example of TIM-3 expression in monocytes and lymphocytes subset. TIM-3 indicates T cell immunoglobulin mucin 3; NK cells, natural killer cells; NKT, natural killer T lymphocytes.

The control group data were obtained from the outpatients’ clinic and patients’ relatives. They were subjected to full history and clinical examination. The mean age was 6.1 (3.7) years, with no history of ITP or autoimmune disorders. Prior to the study, informed consent was obtained from the parents of each participant. All investigations were performed in accordance with the Health and Human Ethical Clearance Committee guidelines for Clinical Research in Assiut University. A flow cytometric detection of lymphocyte and monocyte subsets and their expression of TIM-3 was conducted for both patients and control groups (Figure 1).

### Flow Cytometric Detection of Lymphocyte and Monocyte Subsets and Their Expression of TIM-3

Isolation of PBMCs was performed using Ficoll density gradient centrifugation (Biochrom GmbH, Berlin, Germany). Total of 2×10^6^ cells were stained with 5 µL of 3 panels of antibodies: panel 1 looking for TIM-3 expression on lymphocytes by using fluoroisothiocyanate (FITC)-conjugated anti-TIM-3, phycoerythrin (PE)-conjugated anti-CD8, peridinium-chlorophyll-protein (Per-CP)-conjugated anti-CD4, and allophycocyanin (APC)-conjugated anti-CD3. Panel 2 looking for TIM-3 expression on NK cells and NKT cells by using FITC-conjugated anti-TIM3, Per-CP-conjugated anti-CD56, PE-conjugated anti-CD16, and APC conjugated anti-CD3. Panel 3 looking for TIM-3 expression on monocytes by using FITC-conjugated anti-TIM-3, Per-CP-conjugated anti-CD14, and PE-conjugated anti-CD16. All monoclonal antibodies were purchased from Becton Dickinson (BD) Bioscience, California, except anti-TIM3 which was obtained from Biolegend GmbH, Berlin, Germany.

The cells were washed with phosphate buffer saline (PBS) after incubation for 20 minutes at room temperature in the dark, then resuspended in PBS, and analyzed by FACSCalibur flow cytometry with CellQuest software (BD Biosciences). Antihuman IgG was used as an isotype-matched negative control for each sample. Forward and side scatter histogram was used to define the monocyte and lymphocyte populations. The expression of CD3, CD16, and CD56 were assessed in the lymphocyte population to detect NKT cells (CD3^+^ CD16^+^ CD56^+^), CD3^−^ CD16^+^ CD56^+^^high^ NK cells, and CD3^−^ CD16^+^ CD56^+dim^ NK cells. The CD3^+^ cells (T lymphocytes) were then gated and the expression of CD4 and CD8 on the T lymphocytes were assessed to detect CD4^+^ (T-helper cells) and CD8^+^ (T-cytotoxic cells) cells.

The expression of CD14 and CD16 were analyzed on monocyte population to detect the classical monocytes (CD14^++^ CD16^−^), intermediate monocytes (CD14^+^ CD16^+^), and nonclassical monocytes (CD14^−/dim^ CD16^+^). The expression of TIM-3 was assessed on CD4^+^ cells (TIM-3^+^ CD4^+^), CD8^+^ cells (TIM-3^+^ CD8^+^), nonclassical monocytes (TIM-3^+^ CD14^−/dim^ CD16^+^), intermediate monocytes (TIM-3^+^ CD14^+^ CD16^+^), classical monocytes (TIM-3^+^ CD14^++^ CD16^−^), CD56^+high^ NK cells (TIM-3^+^ CD3^−^ CD16^+^ CD56^+^^high^), CD56^+dim^ NK cells (TIM-3^+^ CD3^−^ CD16^+^ CD56^+dim^), and NKT cells (TIM-3^+^ CD3^+^ CD16^+^ CD56^+^). The expression was reported as percentage of each cell population.

### Statistical Analysis

Data were analyzed using Statistical Package for Social Science version 18.0.A. One-way analysis of variance was applied to find out the significant differences between groups, and the Tukey post hoc test was used to see the main differences among 3 groups. For comparisons between patients and the controls, the independent sample *t* test was used. Data were represented as means (SD). The probability value of < .05 was considered statistically significant.

## Results

The demographic and clinical characteristics of all patients (n = 45) and the controls (n = 20) are summarized in Table 1. The patients’ platelet counts ranged from 9 × 109/L to 39 × 10^9^/L and the mean platelet count was significantly reduced in patients with ITP compared to control participants (*P* = .0001). As regards patient subgroups, significant lower mean platelet count was recorded in the severe bleeding (n = 8) group (11.75 [2.9]) compared to mild (n = 12) bleeding group (32.5 [3.8]) and moderate (n = 25) bleeding groups (23.4 [2.9]; *P =* .0002).

**Table 1.** Baseline Characteristics of Patients With ITP and the Normal Controls.^a^

Parameter	Controls, n = 20	Patients With ITP, n = 45	*P* Value
Age, years	6.1 (3.7)	5.7 (3.4)	.21
Sex, F/M	9/11	21/24	.14
Active bleeding	–	45/45	
Splenomegaly	–	0/45	
Hepatomegaly	–	0/45	
Platelet count, ×10^9^/L	231.9 (42.4)	23.77 (7.5)	<.001
WBC count, ×10^9^/L	7.2 (1.6)	9 (3.9)	·06
Hemoglobin, g/dL	11.1 (0.9)	11.6 (1.2)	.16
MPV, fL	9.4 (1.6)	7.2 (5)	·04

Abbreviations: F/M, female/male; ITP, immune thrombocytopenia purpura; MPV, mean platelet volume; WBC, white blood cells.

^a^Independent sample *t* test. Data represented as mean (SD). *P* ≤ .05 is significant.

### Monocyte and Lymphocyte Subsets in Patients With ITP and the Control Groups

Monocyte and lymphocyte subsets in patients with ITP and the control groups are shown in Table 2. Classical monocytes, CD56^dim^ NK, and T-helper lymphocytes were significantly lower in patients with ITP compared to the controls. In contrast, nonclassical monocytes, intermediate monocytes, NKT, and CD56^high^ NK were significantly expanded upon patients with ITP versus the control group. Finally, between the patients and the controls, there was no significant difference in the cytotoxic T cells.

**Table 2.** Monocyte and Lymphocyte Subsets in Patients With ITP and the Normal Controls.^a^

Parameter	Control, n = 20	ITP, n = 45	*P* Value
Classical monocytes	84.39 (8.78)	77.68 (9.16)	_._008
Intermediate monocytes	5.78 (1.49)	9.82 (1.270	<.001
Nonclassical monocytes	6.87 (1.52)	11.31 (2.32)	<.001
T-helper lymphocytes	49.13 (3.69)	42.48 (9.670	.004
Cytotoxic T lymphocytes	24.64 (4.07)	28.26 (8.23)	.067
NKT lymphocyte	6.42 (1.76)	8.46 (2.80	.005
CD56^high^ NK cells	8.44 (3.89)	20.33 (8.870	<.001
CD56^dim^ NK cells	90.94 (4.77)	79.8 (9.049)	<.001

Abbreviations: ITP, immune thrombocytopenia purpura; NK, natural killer cells; NKT, natural killer T lymphocyte.

^a^Independent sample *t* test. Data represented as mean (SD). *P* ≤ .05 is significant.

### Monocyte and Lymphocyte Subsets Among Different Patient Subgroups

As shown in Table 3, there was a significant difference between different patient subgroups regarding T-helper lymphocytes. Tukey post hoc test revealed that T-helper lymphocytes declined significantly in patients with severe bleeding compared to mild and moderate bleeding groups. In contrast, CD56^high^ NK cells were observed to be significantly higher in severe bleeding group. Cytotoxic T lymphocytes, classical monocytes, intermediate monocytes, nonclassical monocytes, NKT cells, and CD56^dim^ NK cells showed no significant changes among the different diseased groups. In addition, there was no significant changes in all studied cell types between mild and moderate bleeding groups.

**Table 3.** Monocyte and Lymphocyte Subsets Among Subgroups of Patients With ITP.^a^

Parameter	Mild Bleeding, n = 12	Moderate Bleeding, n = 25	Severe Bleeding, n = 8	*P* Value
Classical monocytes	82.16 (526)	75.72 (9.428)	77.125 (11.44)	_._123
Intermediate monocytes	9.87 (1.54)	9.814 (1.22)	9.78 (1.12)	.986
Nonclassical monocytes	10.57 (2.60)	11.55 (2.60)	11.65 (2.74)	.446
T-helper lymphocytes	45.48 (11.9)	43.78 (6.75)	33.95 (10.19)	.016
Cytotoxic T lymphocytes	24.77 (9.31)	27.17 (5.76)	32.61 (8.640	.074
NKT lymphocyte	8.66 (3.32)	8.53 (2.73)	7.93 (2.95)	.846
CD56^high^ NK cell	14.09 (7.69)	20.86 (6.70)	28.04 (10.58)	<.001
CD56^dim^ NK cell	79.37 (10.51)	79.05 (8.37)	82.77 (9.39)	.600

Abbreviations: ITP, immune thrombocytopenia purpura; NK, natural killer cells; NKT, natural killer T lymphocyte.

^a^One-way analysis of variance test. Data represented as mean (SD). *P* ≤ .05 is significant.

### Expression of TIM-3 on Lymphocyte and Monocyte Subsets in Patients With ITP and Control Group

The expression of TIM-3 on CD56 ^high^ NK, T-helper lymphocytes, NKT lymphocytes, and nonclassical monocytes was significantly lower in patients with ITP compared to the controls. In contrast, between patients with ITP and the controls, no significant difference in TIM-3 expression was observed in CD56^dim^ NK cells, cytotoxic T lymphocytes, classical monocytes, and intermediate monocytes (Table 4).

**Table 4.** Expression of TIM-3 on Lymphocyte and Monocyte Subsets in Patients With ITP and Normal Controls.^a^

Parameter	Controls (n = 20)	ITP (n = 45)	*P* Value
TIM-3^+^ CD56^high^ NK cells	50.76 (8.73)	36.97 (10.72)	<0.001
TIM-3^+^ CD56^dim^ NK cells	79.40 (10.47)	74.97 (7.91)	.065
TIM-3^+^ NKT lymphocyte	32.17 (8.52)	30.12 (8.46)	.007
TIM-3^+^ cytotoxic T lymphocytes	5.43 (2.55)	4.82 (1.83)	.273
TIM-3^+^ T-helper lymphocytes	4.80 (1.64)	2.80 (1.36)	.001
TIM-3^+^ classical monocytes	47.99 (19.97)	53.54 (16.94)	.254
TIM-3^+^ intermediate monocytes	37.17 (8.53)	35.59 (6.55)	.416
TIM-3^+^ nonclassical monocytes	46.14 (12.01)	25.65 (8.92)	<.001

Abbreviations: ITP, immune thrombocytopenia purpura; NK, natural killer cells; NKT, natural killer T lymphocyte; TIM-3, T-cell immunoglobulin mucin 3.

^a^Independent sample *t* test. Data represented as mean (SD). *P* ≤ .05 is significant.

### Expression of TIM-3 on Lymphocyte and Monocyte Subsets Among Different Patient Subgroups

We found that TIM-3 expression on CD56^high^ NK cells was significantly different between different bleeding groups, with the lowest level in patients with severe bleeding group compared to the moderate and mild bleeding groups as revealed by Tukey post hoc test. Also, the expression of TIM-3 on NKT lymphocyte, T-helper lymphocytes, and nonclassical monocytes were significantly decreased in the severe bleeding group compared to the moderate and mild bleeding groups (Table 5). Expression of TIM-3 on the classical monocytes, intermediate monocytes, CD56^dim^ NK cells, and cytotoxic T lymphocytes showed no significant changes among the different diseased groups. In addition, there was no significant change in the expression of TIM-3 in all studied cell types between mild and moderate bleeding groups.

**Table 5.** Expression of TIM-3 on Lymphocyte and Monocyte Subsets Among Subgroups of Patients.^a^

Parameter	Mild Bleeding (n = 12)	Moderate Bleeding (n = 25)	Severe Bleeding (n = 8)	*P* Value
TIM-3^+^ CD56^high^ NK cells	41.77 (8.44)	39.54 (7.58)	21.75 (9.25)	<.001
TIM-3^+^ CD56^dim^ NK cells	72.57 (8.37)	76.12 (7.71)	74.98 (8.09)	.454
TIM-3^+^NKT lymphocyte	32.12 (8.08)	31.84 (7.68)	21.74 (6.93)	.006
TIM-3^+^ cytotoxic T lymphocytes	5.90 (2.15)	4.63 (1.53)	3.97 (1.54)	.075
TIM-3^+^T-helper lymphocytes	3.41 (1.28)	2.83 (1.30)	1.81 (1.25)	.033
TIM-3^+^ classical monocytes	49.62 (19.53)	53.23 (15.56)	60.21 (17.27)	.399
TIM-3^+^ intermediate monocytes	34.63 (6.898)	35.303 (6.29)	37.92 (7.15)	.528
TIM-3^+^nonclassical monocytes	31.90 (8.67)	24.03 (6.70)	21.37 (11.47)	.011

Abbreviations: NK, natural killer cells; NKT, natural killer T lymphocyte; TIM-3, T-cell immunoglobulin mucin 3.

^a^One-way analysis of variance test. Data represented as mean (SD). *P* ≤ .05 is significant.

## Discussion

The T-cell immunoglobulin mucin 3 is a type I transmembrane protein that has been implicated in both activation and inhibition of immune responses.^36,37^ Inhibition of TIM-3 pathway in vivo was found to be associated with increased clinical and pathological severity of many Th1-dependent autoimmune diseases.^20^ It was found to be an important negative regulator of Th1-cell activation.^28,29^

The CD56^high^ NK subset is considered an immature NK cells^38,39^ and their expansion was demonstrated in several diseases including patients with multiple sclerosis. The NK cells from these patients inhibited T-cell proliferation and were considered as regulating T-cell proliferation.^40^ An increased proportion of CD56^high^ NK cells were also observed in patients with systemic lupus erythematosus,^41^ hepatitis C virus female, and cytomegalovirus infection.^42^ In the present study, we had demonstrated a high frequency of CD56^high^ NK cells in patients with ITP, and the highest level was found in those patients with severe bleeding. It is not clear whether the expansion of CD56^high^ NK is due to their release in high number (from bone marrow and or lymph nodes) to replace the CD56^dim^ NK cells, which reduced in our patients or due to their involvement in disease pathogenesis. On the other hand, CD56^dim^ NK subset frequency was significantly reduced in patients with ITP in this study, with no significant difference observed between different patient subgroups. Low frequency of CD56^dim^ NK in patients with ITP has been postulated to have a prognostic significance by Talaat el al^12^ who demonstrated a low percentage of CD56^dim^ NK in patients with ITP with a maximum reduction in acute form of the disease.

The T-cell immunoglobulin mucin 3 protein is expressed essentially in all mature CD56^dim^ NK. Previous studies suggested that TIM-3 is a maturation marker on NK cells which are fully cytotoxic and have the maximal cytokine production. However, when TIM-3 was cross-linked to antibodies it suppressed NK-cell-mediated cytotoxicity.^43^ In our study, TIM-3 expression in CD56^high^ NK cells was found to be decreased in patients with ITP and the lowest level was observed in those with severe bleeding. This finding suggested a direct relation between TIM-3 expression and the disease severity.

The NKT cells are a minor leukocyte subset. Their dysfunction or deficiency has been shown to lead to the development of autoimmune diseases and cancer. The NKT cells from patients with ITP were observed to inhibit the proliferation of CD4^+^ T cells in vitro, which may indicate a protective role of NKT cells in ITP.^44^ Ho et al^45^ demonstrated that isolated V24 invariant (V24i) CD1d-restricted NKT, a specific subset of NKT cells, was able to kill antigen-presenting cells, suggesting a mechanism by which NKT cells limit T-cell activation and support the protective role of NKT cells in ITP. In our study, the percentage of NKT cells increased in patients with ITP compared to the controls, while its TIM-3 expression was found to be decreased. On these cells, TIM-3 level was significantly decreased with increasing ITP severity, with the lowest level being observed in severe bleeding group. This finding supports the hypothesis that TIM-3 may downregulate the activity of NKT and alter its protective role in ITP.

The CD4^+^ cells are a type of T cell that plays an important role in the immune system. These cells help to suppress or regulate the immune responses. They divide rapidly once activated and secrete cytokines that regulate or involve in the active immune responses.^15^ The CD8^+^ activation requires help from CD4^+^ T cells, which directly stimulate cytotoxic T-lymphocyte responses.^46^ Shan et al^47^ evaluated the expression of TIM-3 in CD4^+^ T cells in patients with active ITP, patients with ITP in remission, and in healthy participants. They demonstrated decreased levels of TIM-3 expression within the CD4^+^ T cells during active ITP. Additionally, the messenger RNA levels of TIM-3 were found to be significantly decreased in PBMCs during the active stages of the ITP.^48^ In the present study, patients with ITP showed a significant decrease both in CD4^+^ frequency and CD4^+^ TIM-3 expression. Such reduction increased with increasing disease severity, suggesting a possible contribution of TIM-3 expression in ITP pathogenesis and severity.

Human monocytes were shown to trigger T-helper responses during infection and in autoimmune diseases.^49,50^ Nonclassical monocytes produce higher TNF after stimulation and expand under infectious or inflammatory conditions.^18^ Nonclassical monocytes in ITP promote Th1 development, which in turn negatively regulates interleukin 17 and regulatory T-cell induction. This cell may be a critical target in patients with ITP to control inflammatory responses.^51^ The nonclassical subset was negatively correlated with the platelet counts. It had high capability of promoting platelet-reactive T-cell proliferation and promoted the secretion of IFN in patients with ITP.^52^ We observed a significant expansion of the circulating nonclassical monocytes that was associated with significant reduction in their TIM-3 expression. Disease severity was demonstrated to be inversely related to TIM-3 expression as the lowest population of TIM-3 was recorded in the severe bleeding group. No significant difference in cell frequency was reported among patient subgroups, suggesting that TIM-3 expression in nonclassical monocytes may play a role in the regulation of cellular activity rather than cell expansion in patients with ITP.

## Conclusion

The data above collectively revealed relation between the level of TIM-3 expression in PBMCs and the severity of ITP in children, which suggested that TIM-3 may be involved in the pathogenesis of ITP and may be a prognostic marker of disease severity. Restoration of TIM-3 may represent a new therapeutic strategy for ITP in children.
